# Liver transcriptome dynamics in Holstein cows during the periparturient transition

**DOI:** 10.1038/s41598-026-46925-9

**Published:** 2026-04-04

**Authors:** Nayan Bhowmik, Mariana da Silva, Alejandro Castaneda, Asha M. Miles, Congjun Li, Curtis P. Van Tassell, Ransom L. Baldwin, J. Eduardo Rico, George E. Liu

**Affiliations:** 1 Animal Genomics and Improvement Laboratory, Agricultural Research Service, United States Department of Agriculture, Beltsville Agricultural Research Center, Beltsville, MD 20705 USA; 2 Department of Animal and Avian Sciences, University of Maryland, College Park, MD 20742 USA; 3 Department of Clinical Studies, School of Veterinary Medicine, University of Pennsylvania, Kennett Square, Philadelphia, PA 19348 USA; 4 Dairy Records Management Systems, North Carolina State University, Raleigh, NC 27603 USA

**Keywords:** Gene expression, Holstein, Periparturient period, Weighted Gene Co-expression Network Analysis (WGCNA), Genetics, Molecular biology, Physiology

## Abstract

During the periparturient phase, dairy cows undergo profound physiological, metabolic, and immunological changes that affect their health, productivity, and reproductive efficiency, with the liver playing a central role in adaptation. In this study, liver transcriptomic changes were assessed in six multiparous Holstein cows by targeting samples approximately 21 d before expected calving date (D-21) and 7 d after calving (D+7) using RNA sequencing. A total of 198 differentially expressed genes (DEGs) were identified between D-21 and D+7 (140 upregulated and 58 downregulated) using |log_2_[fold-change (FC)]|> 1 and a Benjamini-Hochberg-adjusted *P*-value < 0.05. At D+7, genes associated with lipid handling, energy metabolism, gluconeogenesis, and cell division were activated, reflecting the elevated energy demands of early lactation. In contrast, genes involved in glycogen, polysaccharide, and ketone metabolism were repressed, indicating metabolic prioritization of energy production overgrowth and storage. KEGG pathway analysis revealed activation of endocrine, digestive, and metabolic pathways, while the p53 signaling pathway controlling the cell cycle and DNA damage response was downregulated, likely promoting cell survival and adaptation. Co-expression network analysis further confirmed activation of these biological processes and pathways. In conclusion, these findings provide insights into liver transcriptomic adaptations that enable periparturient Holstein cows to meet the heightened metabolic demands of early lactation, offering potential targets for improving health and productivity during this critical period.

**Supplementary Information:**

The online version contains supplementary material available at 10.1038/s41598-026-46925-9.

## Introduction

The period surrounding calving, specifically three weeks before to three weeks after, is one of the most critical times for highly productive dairy cows. The periparturient period has a significant impact on the health, production, and reproduction of cows and their calves. During this transition phase, cows face significant physiological, metabolic, and immunological challenges. Immunosuppression due to negative energy balance around calving can make cows more susceptible to periparturient infectious diseases and metabolic disorders^1^. These conditions can adversely affect the following lactation and increase the risk of mortality. Furthermore, negative energy balance (NEB) and excessive fat mobilization driven by increased energy demands for fetal growth and milk production, concomitant with reduced feed intake, can lead to increased oxidative stress. Oxidative stress, in turn, compromises the immune system, heightening the risk of infectious diseases in dairy cattle^2^. The intense breakdown of adipose tissue releases free non-esterified fatty acids (NEFA) and oxylipids into the bloodstream, which influence inflammatory responses^3^. Moreover, excessive mobilization of fat and muscle tissue, particularly in the form of NEFA and glucogenic amino acids, during this period negatively affects reproductive performance in dairy cattle^4^. Several hormonal changes occur during this period, including changes in glucocorticoids and reproductive steroids (e.g., increased cortisol around calving, a sharp decline in progesterone before calving, and rising estradiol approaching parturition), together with changes in insulin dynamics and insulin sensitivity, and the periparturient steroid milieu also comprises additional steroid classes, including androgens, as characterized in targeted steroid profiling studies^5–10^. Additionally, there is a decrease in insulin-like growth factor 1 (IGF-1) after parturition^9^. Together, these hormonal shifts significantly influence the metabolic state and overall health of dairy cows. Consequently, understanding these dynamics is crucial for developing effective management and nutritional strategies that mitigate health risks, optimize performance, and enhance the resilience of dairy cows during this critical period.

The liver plays a central role in regulating various metabolic processes, including gluconeogenesis, energy production, lipid and fatty acid metabolism, hormone regulation, stress responses, and inflammatory pathways. During this transition period, the liver is responsible for producing glucose from propionate, lactate, and glucogenic amino acids through gluconeogenesis^11^. Additionally, the liver receives a significant influx of NEFA from mobilized body fat, which leads to liver oxidative metabolism to meet the increased energy demands associated with milk production^12^. During periods of NEB and nutritional deficiency, the expression of the liver’s growth hormone receptor (GHR) is reduced, resulting in decreased synthesis of IGF-1 by the liver, which impacts growth and metabolic regulation^13^. The liver can also experience endoplasmic reticulum (ER) stress due to cellular stress, which may trigger the unfolded protein response to restore proper protein folding and function. This response contributes to conditions such as fatty liver or ketosis^14^. Furthermore, cytokines and acute-phase proteins that mediate inflammatory responses in the liver are essential for the metabolic adaptations that occur during this period^15^.

Several studies have previously analyzed liver biopsy data using microarrays or RNA sequencing to explore the impact of various factors on liver metabolism in periparturient dairy cows. These factors include rumen-protected niacin, nicotinamide, and choline supplementation during the periparturient period^16,17^, dietary phosphorus deprivation during the last 4 weeks of the dry period^18^, subacute ruminal acidosis during the periparturient period^19^, body condition score, and feeding management during the prepartum period^20^. Earlier studies have focused on the impact of dietary energy, parity, and age of cows on changes in liver transcriptomes^21–23^. While previous liver transcriptomic studies have examined the transition period in dairy cows, many have used limited sampling windows and focused primarily on differential expressions. Here, we characterized liver gene-expression changes in U.S. Holstein cows using biopsies collected 21 d prior to expected calving date and 7 d after calving under a clearly defined management and dietary context. We employed a complementary analytical framework combining DESeq2-based differential expression with Weighted Gene Co-expression Network Analysis (WGCNA) to identify both individual differentially expressed genes and coordinated gene co-expression modules associated with the periparturient transition. It is important to note that genotype-by-environment (G × E) interactions are well documented in dairy cattle and can influence biological and performance responses across various production settings. As such, transcriptomic signatures identified under one set of environmental conditions may not directly apply to another context^24–26^. Therefore, we aimed to profile liver transcriptomic differences between D−21 and D+7 in U.S. Holstein cows and to identify gene- and network-level patterns associated with the periparturient transition that can be prioritized for validation in future studies.

## Materials and methods

### Animals and management

**Fig. 1:**
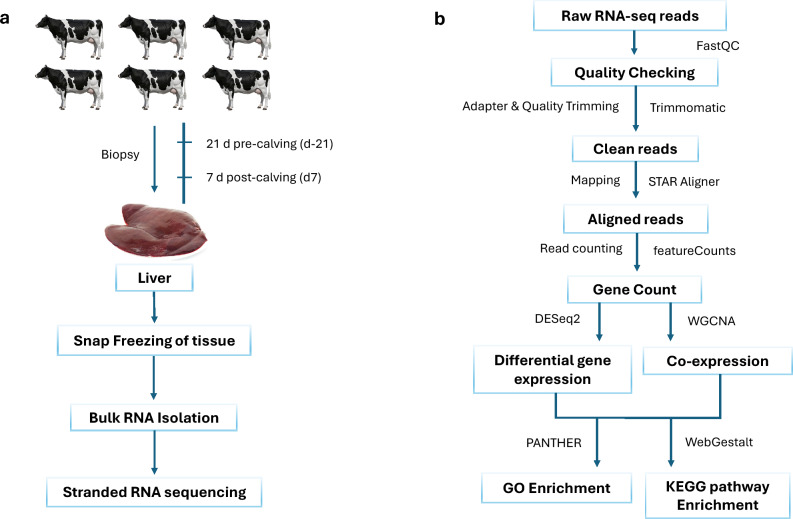
Experimental design. (**a**) Experimental flow chart. (**b**) Data analysis workflow.

The animal study was conducted at the USDA ARS Beltsville Agricultural Research Center (BARC) dairy unit in Beltsville, MD. The animal study protocol was approved by the BARC Institutional Animal Care and Use Committee (IACUC, #AUP-22-01). All methods were carried out in accordance with relevant guidelines and regulations, and all methods are reported in accordance with the ARRIVE guidelines (https://arriveguidelines.org). Liver biopsies were collected from six multiparous (four 2nd lactation cows, one 3rd lactation, and one 4th lactation cow) Holstein cows with a prior mean milk production of 12,304 ± 1430 kg 305 d corrected milk with 4.05 fat and 3.1 protein yields. Liver biopsy sampling targeted 21 d before expected calving date (D-21; 14.8 ± 4.6 d actual prior to calving) and 7 d after calving (D+7; 8.5 ± 1.6 d actual post calving; Fig. 1a). The cows were housed in a ventilated, enclosed freestall barn, where veterinarians and staff daily monitored their health status during the transition period. Recorded health observations included clinical signs of metabolic or infectious disorders (e.g., ketosis, milk fever, mastitis), as well as general indicators of well-being. During the non-lactating phase, cows were maintained on a dry period total mixed ration (TMR) balanced according to NASEM (2021) guidelines^27^ to ensure appropriate nutrient intake during the late gestation (Table 1). Each cow received this specific ration for an average period of 51.3 ± 8.3 d prior to calving. Upon entering the lactation cycle after parturition, the cows were immediately transitioned onto a new lactation TMR specifically optimized for lactating dairy cows, similarly developed in accordance with the NASEM (2021) standards^27^ to support the metabolic demands of early milk production. Table 1 presents the dietary composition, including ingredient inclusion levels on both an as-fed and dry matter (DM) basis.

**Table 1:** Total mixed rations supplied to cows during the dry and lactating period.

Ingredient	As Fed^1^		DM Fed^2^	
	kg/hd^3^	% of ration^4^	kg/hd	% of ration
Dry period				
Dry cow concentrate^5^	2.92	7.16	2.59	15.89
BMR CS^6^	21.53	52.73	6.46	39.55
Wheat straw	4.93	12.08	4.54	27.78
Triticale	8.84	21.65	2.74	16.78
Water	2.61	6.39	0	0
Lactating period				
Milk cow prebatch	18.74	34.55	16.33	65.45
Milk cow concentrate^7^	13.53	70.27	11.91	70.95
Wheat straw	0.89	4.61	0.82	4.86
Grass hay	0.56	2.93	0.51	3.03
Alfalfa	0.57	2.96	0.51	3.05
Cotton seed 2/23	1.36	7.07	1.22	7.30
Citrus pulp 2/23	1.36	7.07	1.22	7.30
Beltsville blend	0.98	5.10	0.59	3.51
BMR CS	22.68	41.82	6.80	27.27
Triticale	5.85	10.79	1.81	7.27
Water	6.96	12.84	0.00	0.00

^1^As Fed refers to the weight of feed offered in its natural moisture state.

^2^DM Fed denotes the corresponding dry-matter basis.

^3^ kg/hd denotes kilograms per head per day.

^4^% of ration values are calculated based on the proportion of total dietary dry matter contributed by each ingredient. The reported values represent formulated rations rather than individually measured intake.

^5^Dry Cow Concentrate – Formulated to meet or exceed dry cow nutrient requirements with a body weight of 581 kg and body condition score of 3.0, following NASEM (2021) guidelines^27^.

^6^BMR CS – Brown Mid Rib Corn Silage.

^7^Milk Cow Concentrate – Formulated to meet or exceed the nutrient requirements of a 2nd lactation Holstein cow weighing 600 kg with a body condition score of 2.5, producing 41 kg milk, following NASEM (2021) guidelines^27^.

### Liver biopsy sample collection

After brushing, clipping, and cleaning the biopsy site ~ 9 cm below the spinal processes on the right lateral of the cow, the area was anesthetized with lidocaine. A 1 to 2 cm incision was made in the 10th intercostal space, and biopsies (~ 1 to 2 g) were obtained by introducing a sterile stainless-steel trocar with a cannula through the incision. The biopsy samples were snap-frozen in liquid nitrogen and stored at -80 °C until they could be shipped for RNA extraction.

### RNA isolation, library construction, and sequencing

All frozen liver tissue samples (n = 12) were shipped to Dante Labs Inc. for RNA extraction and sequencing. Total RNA was extracted from liver tissue by the sequencing service provider (Dante Genomics) as part of the contracted RNA-seq service, according to the provider’s internal standard protocols. While the specific extraction chemistry/kit is not detailed in their public submission documentation, Dante Genomics indicates that they apply extensive sample quality-control procedures for downstream processing. RNA quantity was assessed using spectrophotometry, and RNA integrity was evaluated using an automated electrophoresis system. All samples met sequencing quality thresholds, and the corresponding quality metrics (e.g., RNA integrity values and concentration ranges) were within acceptable ranges. After verifying the quality and quantity of the RNA, libraries for each tissue sample were prepared using the Illumina Ribo-Zero Plus rRNA Depletion Kit. Each library was sequenced to produce at least 6.5 Gb of RNA-seq data using an Illumina NovaSeq 2 × 150 bp platform.

### Pre-processing of RNA sequence data

After receiving the raw FASTQ files from Dante Labs, the quality of each sample was assessed using FastQC (Fig. S2; https://www.bioinformatics.babraham.ac.uk/projects/fastqc/, last accessed on January 5, 2025). The data analysis workflow is provided in Fig. 1b. Poor-quality reads and adapter sequences were trimmed using Trimmomatic software v.0.39^28^ with the following parameters: ILLUMINACLIP:TruSeq3-PE.fa:2:30:10, LEADING:3, TRAILING:3, SLIDINGWINDOW:4:15, and MINLEN:36. The trimmed reads were then aligned to the Bos taurus reference genome assembly ARS-UCD2.0 using the STAR aligner v.2.7.11b^29^ with default settings.

### Differential gene expression analysis

The featureCounts program from the Subread package was utilized to generate gene-level read counts from the aligned RNA-seq data^30^. Counts per million (CPM) values were calculated from the raw integer counts using the *edgeR* framework solely for low-expression filtering and were not used as input for differential expression analysis. Genes with CPM greater than one in at least two samples were retained for analysis.

Differential gene expression analysis was performed using the DESeq2 version 1.44.0 in R^31^, which models raw count data using negative binomial generalized linear models. Because the same animals were sampled at two time points, a paired experimental design was implemented by including cow identity as a blocking factor. The DESeq2 model was specified as follows:

$${\text{Design }} = \sim {\mathrm{cow}}\_{\text{id }} + {\text{ timepoint}}$$

where *timepoint* represented prepartum day −21 and postpartum day +7. Only cows with complete paired samples at both time points were included in the statistical analysis; one sample was excluded because the corresponding animal lacked a matched sample at the alternate time point, which precludes valid paired inference when cow identity is included as a blocking factor. Differential expressions were assessed using a Wald test, and results were summarized for the contrast of postpartum day +7 versus prepartum day −21. Genes with an adjusted *P* value (Benjamini–Hochberg false discovery rate) ≤ 0.05 and an absolute log_2_ fold change > 1 were considered differentially expressed. A Volcano plot was generated using the *ggplot2* package in R to illustrate the up- and down-regulated DEGs.

The normalization of the count data was performed using DESeq2, which calculates the ratio of each sample’s counts to those of a pseudo-reference sample, defined as the geometric mean across all samples. The median of this ratio for each sample serves as the size or normalization factor for that sample. The normalized counts are obtained by dividing the raw counts of each sample by its corresponding size factor. Normalized counts were used to conduct principal component analysis (PCA) for evaluating global transcriptional patterns and sample clustering across time points, with visualization performed using the ggplot2 package^32^ in R.

The identified DEGs were further investigated for gene ontology (GO) term enrichment and Kyoto Encyclopedia of Genes and Genomes (KEGG) pathway enrichment analyses using PANTHER^33^ and the “enrichKEGG” function of the *clusterProfiler* package^34^ in R, respectively. PANTHER maps submitted genes to UniProtKB representative protein IDs, so gene counts for enriched terms reflect these mapped IDs, not the original gene symbols.

### Co-expression network analysis

The raw count data obtained from featureCounts for both stages (D-21 and D+7) were used to identify co-expressed functional gene modules using the WGCNA v.1.73^35^. Genes with counts below 15 in more than 75% of the samples were excluded from the analysis, as these low-expressed features tend to reflect noise, and correlations based on counts that are mostly zero are not meaningful. The variance stabilization method was applied to obtain normalized counts. Analysis of scale-free topology was conducted using the normalized counts to determine the appropriate soft-thresholding power for network construction. A desired minimum scale-free topology fitting index (R2), along with a low mean connectivity, was used to identify this power. The automatic network was then constructed in a block-wise manner using the normalized counts and identified power to detect modules. A dendrogram was generated to visualize the modules before and after merging, utilizing the “plotDendroAndColors” function within *WGCNA*. The module-trait correlation coefficients were calculated using the “cor” function from the *stats* package in R. The correlation *P*-values for each module were adjusted using the false discovery rate (FDR), and modules with FDR-corrected *P*-values of ≤ 0.05 were considered significant. A heatmap plot of module-trait relationships was created using the “labeledHeatmap” function.

The genes from the significant modules were extracted to perform GO term enrichment and KEGG pathway enrichment analyses, employing PANTHER^33^ and the “enrichKEGG” function of the *clusterProfiler* package^34^ in R, respectively.

## Results

### Quality control and mapping of raw reads

The maximum read length for the raw RNA sequence data was 150 base pairs (bp) in a paired-end format. The original RNA-seq raw data from 12 samples underwent quality assessment, revealing an average of 35,455,451 raw reads (Table S1). The average percentages of unique and duplicate reads were 14.55% (ranging from 5.07 to 29.12%) and 85.45% (ranging from 70.88 to 94.93%), respectively. The average Phred quality score across each base position in the 150 bp raw reads was 36.29. Additionally, the average percentages of raw reads with Q20 and Q30 scores were 99.34 and 85.82%, respectively. The average GC content across all samples was 71% (Table S1).

Quality checking also identified the presence of Illumina sequence adapters in some samples. To address this, Trimmomatic was employed to filter the raw reads for quality and adapter content. A total of 31,911,712 clean reads were obtained, indicating that 90.27% of the reads passed the quality control step (Table S1). The clean reads contained an average of 14.09% (with a range of 4.92 to 29.28%) unique reads and 85.91% (ranging from 70.72 to 95.08%) duplicate reads. The average Phred quality score across each base position in the clean reads increased by 3.81% compared to the raw reads. Furthermore, the average percentage of clean reads with a Q30 score rose substantially to 95.36%, reflecting an increase of 11.12% compared to the raw reads, while the percentage of clean reads with a Q20 score increased by 0.66%. The average GC content in the clean reads was 70% across all samples.

These clean reads were subsequently mapped to the Bos taurus ARS-UCD2.0 reference genome assembly. The overall average mapping rate across 11 samples was 93.21% (Table S2). Of the clean reads, 78.68% mapped to unique locations in the genome, while 14.53% mapped to multiple genomic locations. Notably, one sample had a significantly lower mapping rate of 30.36%, with only 27.62% of its reads uniquely mapped and 2.74% mapping to multiple genomic locations, which was excluded from further analysis. The percentage of reads uniquely mapped to the cattle reference genome ranged from 73.32 to 84.46%. Only uniquely mapped reads and those assigned to annotated regions of the cattle genome were used for downstream analysis.

### Principal component analysis

Principal component analysis (PCA) was conducted using count data that were normalized through a variance stabilizing transformation (VST) method. This analysis aimed to explore the relationships both within and between groups of samples. Principal Component 1 (PC1) accounts for 34% of the variation, while Principal Component 2 (PC2) represents 23%. Both PC1 and PC2 collectively distinguish cows at 7 days post-calving from those at 21 days pre-calving (Fig. S1). The lowly mapped sample and the respective unpaired sample were not shown in the PCA plot (Fig. S1).

### Differentially expressed genes between D-21 and D+7 cows

**Fig. 2:**
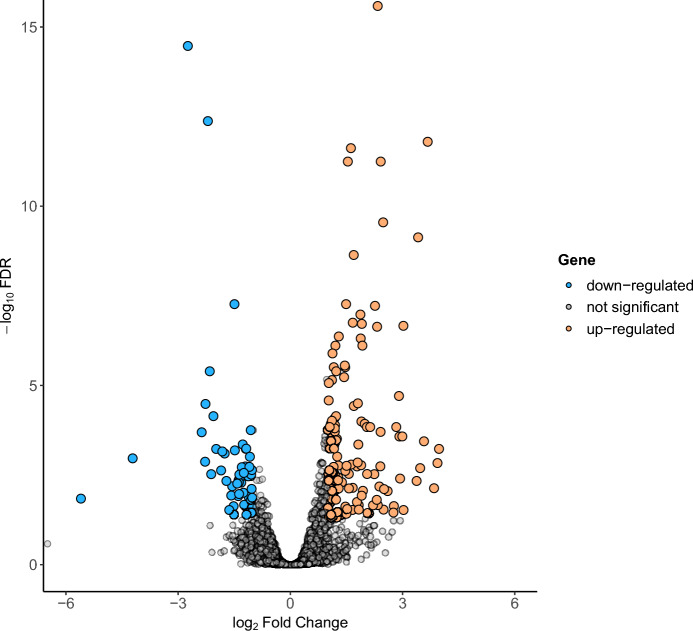
Volcano plot illustrating differentially expressed genes (DEGs) in the liver between 7 days post-calving and 21 days pre-calving.

A total of 22,914 genes with non-zero counts were detected from all samples. After applying a filter for counts per million (CPM) reads greater than one in at least two samples, 13,193 genes were retained for differential gene expression analysis. In total, 198 differentially expressed genes (DEGs) were identified between the cows at day −21 pre-calving (D-21) and day +7 post-calving (D+7), with an adjusted *P*-value of ≤ 0.05 and a |log_2_FC| of ≥ 1. Among these, 140 genes were upregulated, and 58 genes were downregulated in the cows at day +7 after calving (Fig. 2).

Table 2 displays the top 10 upregulated and downregulated genes observed between D+7 and D˗21. A detailed list of DEGs between the pre- and post-partum periods is presented in Table S3. Among the top ten downregulated genes, one is categorized as uncharacterized and shows a significant log_2_-fold change. The top upregulated genes are primarily linked to lipid metabolism and transport, including lipoprotein metabolism and fatty acid/PUFA uptake (*APOA1, MFSD2A, APOA4,* and *ANGPTL4*), energy metabolism (*PDK4*), amino acid metabolism (*HAL*), mitochondrial solute transport (*SLC25A30*), gluconeogenesis (*PC*), cell cycle/mitosis (*CENPF*), and muscle structure (*MYOM1*). Conversely, the top-downregulated genes are involved in carbohydrate metabolism, including glycogen metabolism (*PPP1R3B, PPP1R3C*), innate immunity and defense (*CGN1*), endocrine/vitamin transport (*TTR*), cell signaling (*RAP2C*), cell adhesion and cell–matrix interactions (*FREM3*), receptor/neural-associated signaling (*RTN4RL1*), and translation (*TRNASTOP-UCA*).

**Table 2:** Top DEGs in liver at day +7 post-calving vs day -21 pre-calving, ranked by ascending adjusted *P*-value.

Gene name	Gene symbol	Log_2_ fold change	*P*-value	Adjusted *P*-value
Upregulated				
Apolipoprotein A1	*APOA1*	+2.91	3.59 × 10^–31^	4.74 × 10^–27^
Solute carrier family 25 member 30	*SLC25A30*	+2.34	3.94 × 10^–20^	2.60 × 10^–16^
Major facilitator superfamily domain-containing protein 2A	*MFSD2A*	+3.67	6.05 × 10^–16^	1.60 × 10^–12^
Pyruvate dehydrogenase kinase 4	*PDK4*	+1.62	1.09 × 10^–15^	2.41 × 10^–12^
Apolipoprotein A4	*APOA4*	+2.42	3.28 × 10^–15^	5.69 × 10^–12^
Pyruvate carboxylase	*PC*	+1.54	3.45 × 10^–15^	5.69 × 10^–12^
Angiopoietin-like 4	*ANGPTL4*	+2.48	1.93 × 10^–13^	2.83 × 10^–10^
Centromere protein F	*CENPF*	+3.42	5.61 × 10^–13^	7.40 × 10^–10^
Myomesin 1	*MYOM1*	+1.69	1.92 × 10^–12^	2.30 × 10^–09^
Histidine ammonia-lyase	*HAL*	+1.49	5.32 × 10^–11^	5.40 × 10^–08^
Downregulated				
Conglutinin	*CGN1*	− 2.74	7.71 × 10^–19^	3.39 × 10^–15^
Protein phosphatase 1 regulatory subunit 3C	*PPP1R3C*	− 2.20	1.28 × 10^–16^	4.23 × 10^–13^
Protein phosphatase 1 regulatory subunit 3B	*PPP1R3B*	− 1.49	5.06 × 10^–11^	5.40 × 10^–08^
Uncharacterized LOC112443470	*LOC112443470*	− 2.15	8.58 × 10^–09^	4.03 × 10^–06^
FRAS1-related extracellular matrix protein 3-like	*LOC100337185*	− 2.27	9.21 × 10^–08^	3.28 × 10^–05^
Transfer RNA opal suppressor	*TRNASTOP-UCA*	− 2.06	2.16 × 10^–07^	7.19 × 10^–05^
RAP2C, member of RAS oncogene family	*RAP2C*	− 1.06	6.97 × 10^–07^	1.77 × 10^–04^
Reticulon 4 receptor like 1	*RTN4RL1*	− 2.37	8.75 × 10^–07^	2.02 × 10^–04^
Cortexin domain containing 1	*CTXND1*	− 1.27	2.41 × 10^–06^	4.42 × 10^–04^
Transthyretin	*TTR*	− 1.18	3.31 × 10^–06^	5.79 × 10^–04^

### Gene ontology analysis of DEGs

**Fig. 3:**
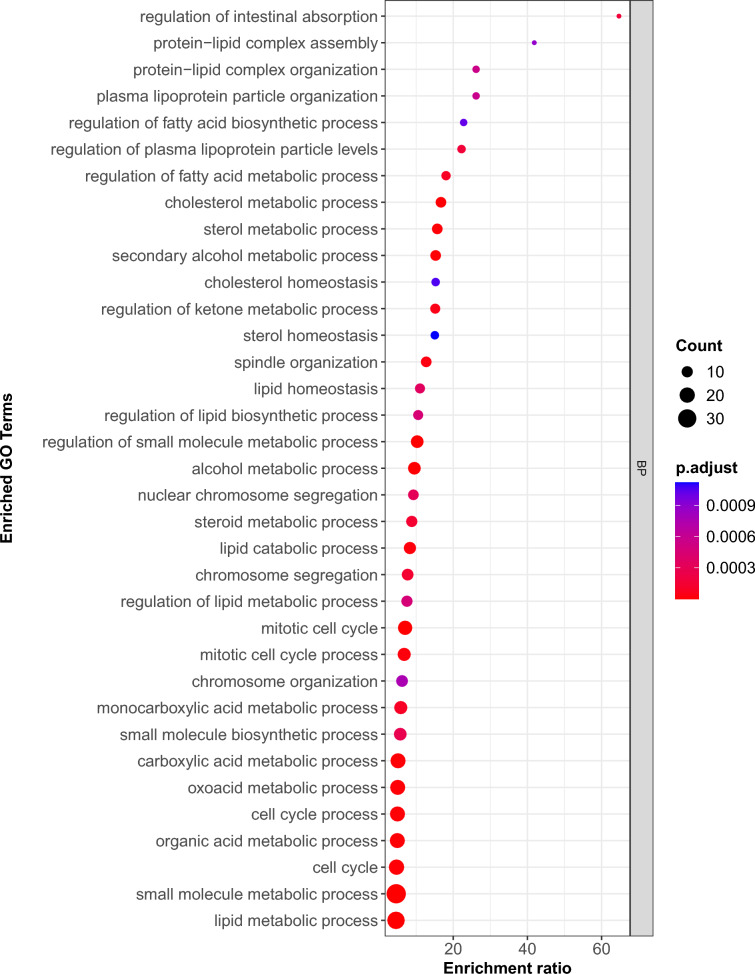
Top 35 enriched GO biological process (BP) terms for the upregulated genes.

We conducted GO enrichment analysis of up- and down-regulated genes separately using PANTHER to gain insights into their biological functions. For the up-regulated DEGs, we identified 122 enriched GO terms, which included only GO terms associated with at least 3 observed gene counts. These GO terms consist of 94 biological processes (BP), 7 molecular functions (MF), and 21 cellular components (CC), as shown in Table S4. Figure 3 illustrates the top 35 GO-enriched biological processes for upregulated genes. Based on GO term definitions, approximately 45.71% of these top biological processes were associated with lipid metabolism/lipid homeostasis/lipoprotein organization. Additionally, 22.86% of the top GO-enriched biological processes were related to cell division and chromosome segregation. About 25.71% of them were linked to general intermediary metabolism. The biological processes directly related to ketone metabolism and digestive system function were also enriched. The biological process “lipid metabolic process” had a fold enrichment score of 4.56 and contained 24 upregulated DEGs (*MGLL, ABCA4, PCK1, ASPG, ABHD1, APOA1, CYP4A59, CYP7A1, ACADVL, GK, LPIN1, APOC2, LIPG, PC, ELOVL5, LEPR, FDFT1, CPT1B, LOC112449293, CYP11A1, APOA4, PLCD4, SOAT2,* and *ANGPTL4*). Moreover, the “sterol homeostasis” biological process had an enrichment score of 15.03 and included six upregulated genes (*APOA1, CYP7A1, LIPG, HSDL2, APOA4,* and SOAT2).

**Fig. 4:**
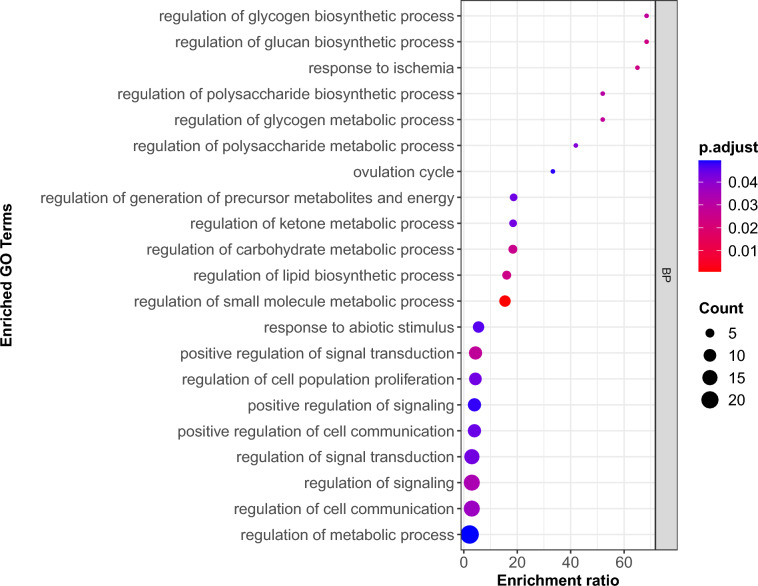
Enriched GO biological process (BP) terms for the downregulated genes.

A total of 58 downregulated genes were analyzed, resulting in the enrichment of 27 GO terms: 21 BP, 2 MF, and 4 CC, as presented in Table S5. The 21 enriched GO BP terms for the downregulated genes are presented in Fig. 4. The GO enrichment of downregulated genes highlighted processes involving i) regulation of metabolism, including carbohydrate/polysaccharide (glycogen/glucan) metabolic regulation (23.21% of enriched BP terms), ketone metabolic regulation, precursor-metabolite/energy-generation regulation; ii) cell communication/signaling and signal-transduction regulation (28.57%); iii) regulation of cell population proliferation; and iv) responses to stress (abiotic stimulus and ischemia), alongside an ovulation-cycle term. The biological process term “regulation of the glycogen metabolic process” had an enrichment score of 52.00 and was linked to three DEGs, namely *PPP1R3C, PPP1R3B,* and *IGF1*.

### KEGG pathway analysis of DEGs

The KEGG pathway analysis was performed using both up-regulated and down-regulated genes to investigate the biological pathways affected during the D+7 period. The results of the pathway enrichment analysis are presented in Table 3, highlighting the most enriched pathways with a *q*-value of less than 0.05. A total of 14 KEGG pathways were identified for the up-regulated genes, while only one pathway was identified for the down-regulated genes. About 59% of the pathways enriched in the upregulated genes were related to metabolism (including carbohydrate, fat, and amino acid metabolism), 24% were associated with organismal systems (such as the endocrine and digestive systems), and 18% were linked to cellular processes (including cell growth and death, cell motility, transport, and catabolism). The three pathways with the highest enrichment scores – 13.45 for fatty acid degradation, 13.02 for the citrate cycle, and 12.01 for the PPAR signaling pathway – were associated with the metabolism of fat and carbohydrates. The pathways related to lipid metabolism included fatty acid degradation, fatty acid metabolism, cholesterol metabolism, peroxisome, and PPAR signaling pathways. For carbohydrate metabolism, the enriched pathways included the citrate cycle, pyruvate metabolism, propanoate metabolism, and glycolysis or gluconeogenesis. Additionally, glucagon and adipocytokine signaling pathways were also enriched. The digestion and absorption of vitamins were also identified. The pathway involving glycine, serine, and threonine metabolism is also upregulated at postpartum. The p53 signaling pathway, which regulates the cell cycle and DNA damage response, was downregulated at D+7.

**Table 3:** List of KEGG pathways enriched in the up- and down-regulated genes.

ID	Category	Name	Count	Fold enrichment	*q*-value	Core enrichment genes
Upregulated						
bta03320	Endocrine system	PPAR signaling pathway	9	16.50	3.91 × 10^–07^	*APOA1, ANGPTL4, CPT1B, GK, CYP4A11, PCK1, RXRG, CYP4A59, CYP7A1*
bta04979	Digestive system	Cholesterol metabolism	7	22.00	1.61 × 10^–06^	*APOA1, APOA4, ANGPTL4, APOC2, LIPG, SOAT2, CYP7A1*
bta04922	Endocrine system	Glucagon signaling pathway	7	10.47	1.88 × 10^–04^	*CPT1B, LDHB, PPARGC1A, PCK1, CREB3L3, LDHA, GYS2*
bta04920	Endocrine system	Adipocytokine signaling pathway	6	12.66	2.37 × 10^–04^	*CPT1B, LEPR, PPARGC1A, PCK1, RXRG, ADIPOR2*
bta04152	Signal transduction	AMPK signaling pathway	7	8.56	4.26 × 10^–04^	*CPT1B, LEPR, PPARGC1A, PCK1, CREB3L3, ADIPOR2, GYS2*
bta00561	Lipid metabolism	Glycerolipid metabolism	5	11.33	1.65 × 10^–03^	*GK, LIPG, LPIN1, MGLL, LOC618076*
bta00071	Lipid metabolism	Fatty acid degradation	4	13.69	3.24 × 10^–03^	*CPT1B, ACADVL, CYP4A11, CYP4A59*
bta00620	Carbohydrate metabolism	Pyruvate metabolism	4	13.69	3.24 × 10^–03^	*PC, LDHB, PCK1, LDHA*
bta04931	Endocrine and metabolic disease	Insulin resistance	5	7.00	1.04 × 10^–02^	*CPT1B, PPARGC1A, PCK1, CREB3L3, GYS2*
bta00020	Carbohydrate metabolism	Citrate cycle (TCA cycle)	3	14.91	1.35 × 10^–02^	*PC, LOC132342072, PCK1*
bta00140	Lipid metabolism	Steroid hormone biosynthesis	4	7.90	1.92 × 10^–02^	*CYP11A1, UGT2B10, CYP7A1, LOC112449293*
bta04814	Cell motility	Motor proteins	6	4.51	2.27 × 10^–02^	*KIF4A, MYO7B, KIFC1, CENPE, KIF20A, KIF15*
bta04936	Endocrine and metabolic disease	Alcoholic liver disease	5	4.87	3.58 × 10^–02^	*CPT1B, ACADVL, PPARGC1A, LPIN1, ADIPOR2*
bta04975	Digestive system	Fat digestion and absorption	3	9.06	4.07 × 10^–02^	*APOA1, APOA4, LOC618076*
Downregulated						
bta04115	Cell growth and death	p53 signaling pathway	4	13.42	2.56 × 10^–02^	*IGFBP3, SESN3, CDKN1A, IGF1*

### Gene co-expression network analysis

**Fig. 5:**
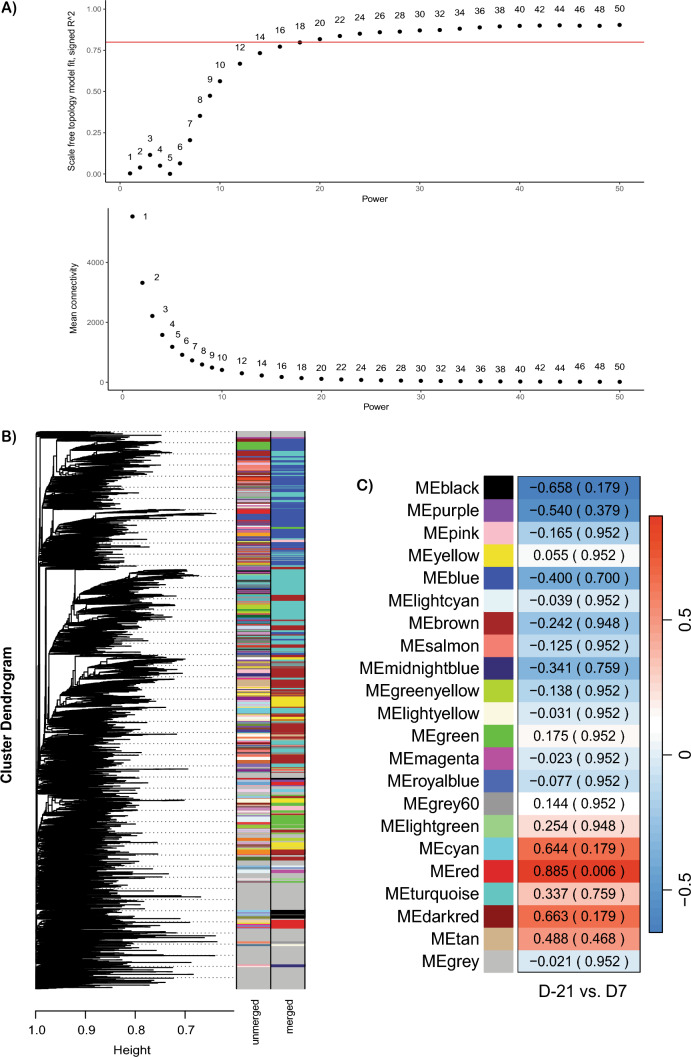
Weighted gene co-expression network analysis (WGCNA). (**a**) Power plot shows scale-free fit index and mean connectivity for various soft thresholding powers. The red line indicates the scale-free topology index value of 0.80. (**b**) Clustering dendrogram of genes with assigned merged and unmerged module colors. (**c**) Heatmap of module-trait association indicating correlations between module eigengenes (22) and periparturient stages, with FDR adjustment.

A co-expression network analysis was conducted using the WGCNA R package^35^ to identify groups of co-expressed genes (modules) that were expressed at different levels in cows seven days postpartum compared to their expression levels during the 21-day prepartum period. We constructed a scale-free co-expression gene network using the one-step network construction method. The network topology analysis was conducted using the “pickSoftThreshold” function. To identify the optimal power, we tested a range of soft threshold values and created two diagnostic plots: one displaying the relationship between power and the scale-free topology model fit (Signed R^2^) indices, and another showing the relationship between power and mean connectivity indices (Fig. 5a). The soft threshold power was chosen to strike a balance between the scale-free topology fit and mean connectivity. We determined that a power value of 20 achieved a higher Signed R^2^ (0.818) while maintaining a relatively low mean connectivity (113). This soft threshold value was then used to construct the network with the “blockwiseModules” function. A total of 22 co-expression modules were identified after merging closely related modules, specifically those with eigengenes that have over 80% similarity (r = 0.8; Fig. 5b).

**Fig. 6:**
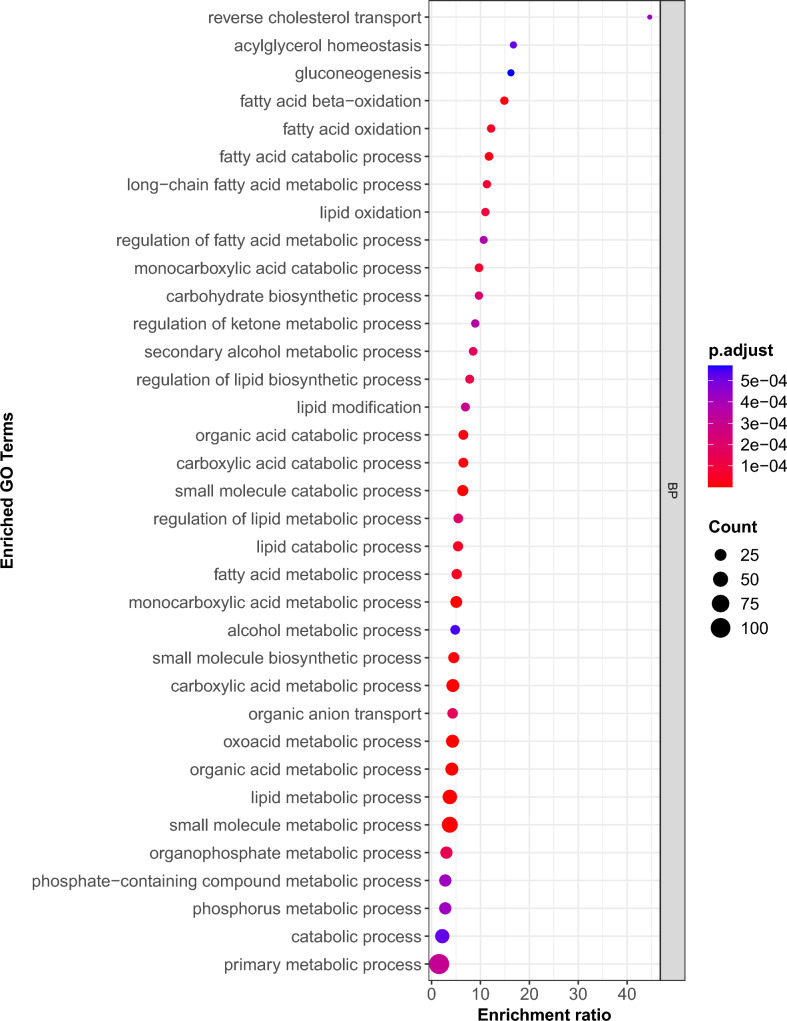
Top 35 enriched GO biological process (BP) terms for the genes in the significant red module.

The module-trait association identified one significant module, labeled “red” (*P*adj = 0.006), out of a total of 22 modules (Fig. 5c). This red module contained 259 hub genes. The hub genes were subsequently used for GO enrichment and KEGG pathway analyses. The GO enrichment analysis identified 162 enriched GO terms, which included 113 BP, 33 MF, and 16 CC, as shown in Table S6. Figure 6 displays the top 35 biological process terms that are enriched in GO. The GO enrichment analysis, along with the significant WGCNA module, further confirmed the activation of biological processes identified with differential gene expression analyses. Approximately 40.00% of these terms were associated with fat metabolism, while 34.29% were linked to general intermediary metabolism, including organic acid/carboxylic acid/oxoacid metabolism. The BP terms involved in carbohydrate, phosphate/phosphorus, ketone, and alcohol metabolism are also enriched. We subsequently performed KEGG pathway enrichment analysis using the 259 hub genes identified in the significant red module from the co-expression network analysis to determine the biological pathways impacted at the 7-day postpartum period. A total of 10 KEGG pathways were activated during this transition in postpartum cows (Table 4). About 7 of these KEGG pathways were also enriched in the differential gene expression analysis. About 80% of these enriched KEGG pathways were associated with lipid metabolism.

**Table 4:** List of KEGG pathways enriched in the genes of the significant red module.

ID	Category	Name	Count	Fold enrichment	*q*-value	Core enrichment genes
bta03320	Endocrine system	PPAR signaling pathway	14	11.57	2.61 × 10^–09^	*EHHADH, ACADL, RXRG, HMGCS2, CPT2, CYP4A11, CYP4A59, ANGPTL4, SLC27A2, APOA1, NR1H3, ACSL1, CPT1A, GK*
bta00071	Lipid metabolism	Fatty acid degradation	9	13.88	1.34 × 10^–06^	*EHHADH, ACADL, CPT2, CYP4A11, CYP4A59, HADHA, ACAA2, ACSL1, CPT1A*
bta04979	Digestive system	Cholesterol metabolism	8	11.33	3.15 × 10^–05^	*SORT1, ANGPTL4, ABCA1, APOA4, APOA1, SCARB1, APOC2, MYLIP*
bta01212	Global and overview maps	Fatty acid metabolism	8	9.41	1.03 × 10^–04^	*EHHADH, ACADL, CPT2, HADHA, ELOVL5, ACAA2, ACSL1, CPT1A*
bta04146	Transport and catabolism	Peroxisome	8	6.61	1.20 × 10^–03^	*EHHADH, IDH1, ABCD3, DHRS4, SLC27A2, MLYCD, AMACR, ACSL1*
bta04152	Signal transduction	AMPK signaling pathway	9	4.96	2.65 × 10^–03^	*LEPR, GYS2, ADIPOR2, PPARGC1A, CREB3L3, FBP1, AKT3, MLYCD, CPT1A*
bta04920	Endocrine system	Adipocytokine signaling pathway	7	6.66	2.65 × 10^–03^	*RXRG, LEPR, ADIPOR2, PPARGC1A, AKT3, ACSL1, CPT1A*
bta04931	Endocrine and metabolic disease	Insulin resistance	8	5.05	5.13 × 10^–03^	*GYS2, PPARGC1A, CREB3L3, SLC27A2, NR1H3, AKT3, PPP1R3C, CPT1A*
bta04977	Digestive system	Vitamin digestion and absorption	4	10.28	1.41 × 10^–02^	*SLC19A3, APOA4, APOA1, SCARB1*
bta04922	Endocrine system	Glucagon signaling pathway	7	4.72	1.58 × 10^–02^	*GYS2, PPARGC1A, CREB3L3, FBP1, SIK2, AKT3, CPT1A*

## Discussion

This study focuses on differentially expressed and co-expressed genes and their metabolic pathways in the liver of periparturient cows, utilizing liver biopsy sampling targeted 21 d before expected calving date (D-21; 14.8 ± 4.6 d actual prior to calving) and 7 d after calving (D+7; 8.5 ± 1.6 d actual post calving). During the dry period, cows were offered a total mixed ration (TMR) formulated to meet or exceed the nutrient requirements for late-gestation cows as established by NASEM (2021)^27^. Upon calving, cows were transitioned to a high-production TMR formulated to meet nutrient requirements for early lactation, in accordance with NASEM (2021) guidelines^27^. Prior to the dry period, cows were maintained on a standard ration formulated for lactating, high-producing cows. As a central organ for metabolism, the liver is substantially influenced by dietary components during the dry period. Dairy cows fed high energy diets at prepartum period exhibited increased lipid accumulation and triglyceride synthesis in the liver, subsequently resulting in greater liver distress after calving^36^. In contrast, cows fed restricted energy diets during the prepartum period exhibited a greater ability to break down lipids and amino acids, which enhanced adaptability when metabolic challenges arise postpartum. In fact, the restricted diet enhanced their capacity to manage ER stress, likely due to increased protein synthesis and processing^36^. A recent study found that prepartum high energy intake activated a transcriptional cascade of events, partly driven by the activation of PPARγ, that regulates preadipocyte differentiation and lipid storage in subcutaneous adipose tissue during the periparturient period in Holstein dairy cows^37^. Likewise, prepartum excessive energy intake is linked to sustained levels of ER stress and oxidative stress postpartum, along with the upregulation of inflammation-related genes in dairy cows^38^. A study on restricted feeding during the prepartum period observed improved capacity for lipid and amino acid metabolism, indicating that feed-restricted cows adapted better to metabolic challenges^20^. Moreover, varying dietary energy levels before the dry period can have lasting effects during early lactation, impacting body condition, metabolic status, performance, and hepatic gene expression^39,40^. These findings demonstrate that different energy diets administered to cows at prepartum period and before the dry period can influence liver transcriptomic profiles during the periparturient period. Prior studies have assessed transcriptomic changes across the prepartum and postpartum periods in dairy cows using diets containing different energy levels or with no information on nutritional management^41,42^. To facilitate interpretation of similarities and differences in experimental design, dietary context, and key transcriptomic findings across major periparturient liver transcriptomic studies, a concise comparison, including the current study, is provided in Table S7. Therefore, our study focused on exploring liver transcriptomic changes in periparturient cows fed both a common dry-period diet and a common lactation diet.

At the gene level, the top upregulated DEGs were associated with lipid metabolism and transport (including lipoprotein metabolism and fatty-acid/PUFA uptake), energy metabolism, gluconeogenesis, amino acid metabolism, and mitochondrial solute transport. The observed upregulation of *APOA1, MFSD2A, APOA4,* and *ANGPTL4* is directly supported by our differential gene expression analysis, while their roles in lipid metabolism and transport are supported by earlier studies. The *APOA1* gene encodes apolipoprotein A1, the major protein component of high-density lipoprotein (HDL), and it plays a central role in reverse cholesterol transport^43,44^. The HDL particles produced by this protein exhibit several beneficial properties, including anti-inflammatory, anti-atherogenic, anti-apoptotic, and anti-thrombotic effects, that occur through various mechanisms. Additionally, HDL plays a crucial role in removing cholesterol from peripheral tissues and transporting it to the liver for excretion via the reverse cholesterol transport mechanism^45^. APOA1 is synthesized predominantly in the liver and also in the intestine, and then it is secreted into the circulation, where it associates with HDL particles^43,44^. The *APOA1* gene has been shown to be upregulated in the liver in several studies: at 2 weeks postpartum in Holstein cows that do not exhibit subacute ruminal acidosis (non-SARA)^19^, and between 10 to 14 days postpartum in cows with ketosis^46^. *APOA1* was also found to be up-regulated in over-conditioned cows with a more balanced metabolic phenotype as compared to over-conditioned cows with an “obese” phenotype at d21 of lactation^40^, where over-conditioned was achieved by divergent feeding before the dry period. The *MFSD2A* gene encodes a sodium-dependent lysophosphatidylcholine transporter expressed at the blood–brain barrier^47^, which transports docosahexaenoic acid (DHA) primarily in the form of lysophosphatidylcholine (LPC-DHA), rather than unesterified DHA^48,49^. This protein is crucial for transporting essential long-chain fatty acids, particularly DHA, to the brain. Hepatic MFSD2A is exclusively expressed in periportal hepatocytes and is induced by fasting in mice^50^. Additionally, it plays a facilitative role in body growth and development, as well as in motor function and lipid metabolism^51^. The *MFSD2A* was found to be upregulated in the adipose tissue of dairy cows at 2 weeks postpartum^52^, in the early postpartum liver of dairy cows comparing multiparous and primiparous cows^23^, and during the late gestation period of protein-supplemented Nelore bulls^53^. Furthermore, the *APOA4* gene encodes apolipoprotein A-IV, a multifunctional apolipoprotein implicated in lipid adsorption and lipoprotein metabolism; in mice, this protein has been shown to promote hepatic triglyceride secretion (including via very low-density lipoprotein [VLDL]) and reduce hepatic lipid accumulation^54^. The APOA4 has also been reported to improve glucose homeostasis, at least in part by enhancing glucose-stimulated insulin secretion^54,55^. An upregulation of the *APOA4* gene was observed at 10 days postpartum in the liver of dairy cows^56^ and at 2 and 6 weeks after parturition in the non-SARA group of Holstein cows^19^ compared to prepartum period. The *ANGPTL4* gene encodes the angiopoietin-like 4 protein, a secreted protein that inhibits lipoprotein lipase (LPL) and thereby regulates plasma triglyceride metabolism and tissue lipid uptake^57,58^. It also influences glucose homeostasis by affecting lipid metabolism and insulin sensitivity, as well as playing a role in angiogenesis^59,60^. Previous studies have indicated that the *ANGPTL4* gene is overexpressed in the livers of postpartum cows experiencing significant NEB^61^. Additionally, mRNA levels of this gene have been found to be abundant in the subcutaneous adipose tissue of dairy cows during early lactation^62^. Interestingly, an upregulation of the *ANGPTL4* gene’s mRNA expression has also been observed in the liver of prepartum cows treated with recombinant bovine somatotropin^63^.

The remaining five of the top ten upregulated DEGs were linked to amino acid metabolism, gluconeogenesis, mitochondrial solute transport, energy metabolism, cell cycle/mitosis, and muscle structure. The histidase enzyme, also known as histidine ammonia-lyase, encoded by the *HAL* gene, is necessary for histidine catabolism, converting L-histidine to trans-urocanic acid. This process facilitates the complete breakdown of histidine into ammonia, glutamate, and other metabolites^64^. An earlier study reported an upregulation of the *HAL* gene in the liver of beef cows at − 15 days relative to the calving period, compared to -165 days relative to the calving period^65^. Pyruvate carboxylase is encoded by the *PC* gene, which is a vital enzyme for gluconeogenesis, and converts non-carbohydrate substrates into glucose. This process helps maintain energy balance and provides substrates for lipogenesis^66^. Multiple studies have investigated the expression of the pyruvate carboxylase gene in the liver of periparturient dairy cows. An increased *PC* gene expression was observed in 14 days postpartum Holstein cows transitioning into ketosis^46^. This gene expression was also upregulated in postpartum Holstein cows without non-subacute ruminal acidosis^19^. An increase in the mRNA levels of the *PC* gene has been observed in the livers of Holstein transition dairy cows infused with 5-hydroxytryptophan^67^. Additionally, this increase was noted in the postpartum livers of multiparous Holstein cows that were fed protein during the prepartum period^68^. Moreover, an enhanced expression of the promoter 1-regulated PC transcript was reported during the early postpartum phase^69^. The *SLC25A30* gene, a member of the mitochondrial carrier family, facilitates cellular energy production by connecting mitochondrial and cytosolic metabolic pathways through the transport of various solutes, including carboxylates, amino acids, nucleotides, and cofactors, across the mitochondrial membrane^70,71^. In early lactation, *SLC25A30* was also identified among hepatic genes contributing to an enriched transport-related functional term when comparing metabolically imbalanced versus balanced cows sampled at approximately 14 DIM^72^. The upregulation of other solute carrier family members, including *SLC12A8* and *SLC16A5*, was previously observed in a related study^40^. *PDK4* encodes pyruvate dehydrogenase kinase 4, which phosphorylates the E1α component of the pyruvate dehydrogenase complex and inhibits conversion of pyruvate to acetyl‑CoA, thereby reducing pyruvate oxidation and supporting metabolic flexibility between glucose and fatty‑acid utilization^73,74^. In early-lactation dairy cows, *PDK4* has been reported among genes associated with metabolic clustering linked to energy balance in the liver and blood leukocytes^72^. *CENPF* encodes centromere protein F (CENP‑F), which accumulates in late G2, localizes to kinetochores during mitosis, and is degraded after mitosis, consistent with its established role in cell-cycle progression and chromosome segregation^75,76^. *MYOM1* encodes myomesin‑1, a major component of the sarcomeric M‑band that cross-links thick filaments and contributes to myofibrillar structural integrity^77,78^. The upregulation of this gene was reported in the liver of peripartum beef cows on a grazing system (day +15 postpartum vs day -15 prepartum cows)^65^. These top upregulated genes found in our study were also reported to be upregulated significantly in the liver of postpartum dairy cows compared to prepartum cows^42^, and five of these genes (*ANGPTL4*, *APOA4*, *MFSD2A*, *MYOM1*, and *PDK4*) were significantly upregulated at day +10 and/or day +17 postpartum relative to day -22 prepartum in red Holstein cows^41^. The increase in gene expression associated with lipid and lipoprotein metabolism, gluconeogenesis, energy metabolism, and amino acid metabolism during the early postpartum period represents a complex metabolic adaptation. This adaptation is primarily driven by NEB, which occurs due to the high energy demands of lactation combined with reduced feed intake^79^.

The top downregulated genes were linked to glycogen metabolism, innate immunity and defense, endocrine/vitamin transport, cell signaling, and neural-associated signaling. The *CGN1* gene encodes bovine conglutinin, a liver-synthesized serum collectin/opsonin that participates in innate host defense by binding deposited iC3b and microbial glycoconjugates^80^. The *PPP1R3B* gene regulates glycogen synthesis in the liver by producing a regulatory subunit of protein phosphatase 1 (PP1). The PP1 enzyme, recruited to glycogen by glycogen-targeting regulatory subunits, promotes glycogen synthesis by dephosphorylating (activating) glycogen synthase and dephosphorylating (inactivating) glycogen phosphorylase^81,82^. *PPP1R3B* was reported to be downregulated in the livers of early lactating cows (10 days postpartum) compared to their dry period (50 days prepartum)^56^. The *PPP1R3C* gene encodes regulatory subunits of protein phosphatase 1, playing a similar role to the *PPP1R3B* gene in promoting glycogen synthesis and storage in the liver^83^. This gene expression was downregulated in the livers of early postpartum cows due to supplementation with grape seed and grape marc meal extracts, which was part of a broader decrease in genes related to ER stress and unfolded protein response (UPR) pathways^84^. However, *PPP1R3C* gene expression was upregulated in the insulin-treated healthy postpartum group (cows with β-hydroxybutyrate [BHBA] < 1.4 mmol/L) compared to the control ketosis group (cows with BHBA ≥ 1.4 mmol/L)^85^. In addition, both the *PPP1RB* and *PPP1R3C* gene expressions were downregulated in the livers of older multiparous cows early in lactation compared to those of primiparous cows^23^. The *RAP2C* gene regulates cell adhesion and migration through the Akt and MAPK/ERK signaling pathways by controlling MMP2 activity and the recycling of adhesion molecules, such as LFA-1^86,87^. The *FREM3* gene, part of the Fras1/Frem family, encodes a protein found in the extracellular matrix. This protein is vital for regulating cell adhesion and maintaining the structural integrity of basement membranes. It achieves this by facilitating the adhesion of epithelial cells to the underlying mesenchyme through the formation of a macromolecular complex with other family members, such as Fras1 and Frem2^88^. *TRNASTOP-UCA* is annotated as a transfer RNA (tRNA) gene; tRNAs are core adaptor RNAs required for decoding mRNA codons during protein synthesis^89,90^. *RTN4RL1* (also known as NgR3/NgRH2) is a GPI-anchored leucine-rich repeat receptor within the Nogo receptor family, which has been implicated in neurite outgrowth inhibition and axon regeneration pathways in the central nervous system^91–93^. *CTXND1* encodes a cortexin-domain containing protein; cortexin proteins were originally described as small membrane proteins enriched in the cerebral cortex, but *CTXND1* itself remains comparatively poorly characterized in the peer-reviewed literature^94^. *TTR* encodes transthyretin, a plasma transport protein synthesized mainly by the liver that carries thyroxine and the retinol-binding protein–retinol complex^95,96^. Our findings align with previous research indicating that *CGN1, PPP1R3B, PPP1R3C, RAP2C,* and *TTR* are significantly downregulated in the livers of postpartum dairy cows compared to prepartum cows^42^. Notably, four of these genes – *PPP1R3B, PPP1R3C, RAP2C,* and *TTR* – were shown to be downregulated in at least one of the comparisons on day +10 or day +17 postpartum when compared to day -22 prepartum in red Holstein cows^41^. The downregulation of the genes related to glycogen metabolism and storage in the liver, immune responses, endocrine transport, and cell signaling is also part of significant physiological and metabolic changes that occur during the transition period, since the body of the cow prioritizes energy allocation for milk production and adaptation to the early lactation stress^16,40,97^.

For GO enrichment and KEGG pathway analysis, we compared our results with previous studies that used RNA sequencing data to investigate the liver adaptation of dairy cows. However, we focused on studies that measured gene expression in periparturient cows subjected to various experimental interventions, such as lactation-induced NEB, supplementation, or special diets, for our discussion. During the early postpartum period, NEB increases lipolysis in adipose tissue and circulating non-esterified fatty acids (NEFA). This leads to increased hepatic uptake of NEFA, and the postpartum liver exhibits greater capacity for fatty-acid beta-oxidation and mitochondrial fatty acid processing compared to the prepartum period^6,98^. In this context, our data showed significant enrichment of various biological processes (e.g., lipid metabolic process, lipid catabolic process, protein-lipid complex organization/assembly, plasma lipoprotein organization/regulation) alongside KEGG pathways including fatty acid degradation, glycerolipid metabolism, and fat digestion and absorption, which are directly associated with lipid handling and energy production. These enriched GO biological process terms and KEGG pathways related to lipid metabolism align with previous transcriptomic studies that noted similar pathway enhancements in the livers of early postpartum cows^16,17,41,42,56,61,99^. These enrichments collectively highlight the liver’s central role in adapting to the energy deficit by enhancing lipid mobilization, uptake, and oxidative catabolism. Our dataset reveals a substantial activation of genes associated with fatty acid metabolism pathways, consistent with increased β-oxidation to support ATP generation during periods of limited glucose availability.

Early lactation imposes a major homeorhetic shift: nutrient requirements (especially for glucose/energy) rise sharply, while cows frequently experience NEB. The liver must rapidly adapt to support lactation and systemic energy homeostasis, and this adaptation is mediated by coordinated endocrine and nutrient-sensing signals. In classic transition cow physiology, hormonal adjustments and changes in insulin function contribute to the normal distribution of nutrients toward the mammary gland while managing negative energy balance^6,98,100^. Consistent with this framework, our study demonstrated high activation of BP terms related to metabolic regulation and homeostasis, including regulation of lipid metabolic process, regulation of fatty acid metabolic process, regulation of lipid biosynthetic process, lipid homeostasis, cholesterol homeostasis, and sterol homeostasis. In parallel, KEGG enrichment also highlighted endocrine/energy-sensing signaling maps that coordinate metabolic state, including AMPK signaling, glucagon signaling pathway, adipocytokine signaling, PPAR signaling, and insulin resistance pathways.

Importantly, the activation of the PPAR signaling pathway provides a regulatory explanation for the lipid-centric liver response. PPARs are lipid-activated nuclear receptors that regulate transcriptional programs involved in lipid metabolism; in ruminant systems, PPARα activation has been linked to increased expression of genes involved in fatty acid transport and oxidation, and postpartum NEFA dynamics have been evaluated as a potential upstream driver of bovine PPAR activity^101,102^. We observed enhanced activation of PPAR signaling pathway genes, consistent with PPARα functioning as a key transcriptional regulator coordinating lipid catabolic programs during the early postpartum stage.

Our study also revealed significant enrichment in cholesterol metabolism, indicating additional layers of liver metabolic adaptation. Cholesterol homeostasis is linked with lipoprotein metabolism and bile acid-dependent lipid digestion. The increased expression of genes such as *APOA1*, *APOA4*, and *ANGPTL4* in our data supports involvement of lipoprotein-associated cholesterol transport processes during early lactation. These findings align with earlier reports that demonstrated the upregulation of the cholesterol metabolic pathway in the liver of periparturient cows^17,41,56^, and increased liver transcription of apolipoprotein genes during early lactation^103^, consistent with altered lipid flux. Activation of the adipocytokine signaling pathway, particularly through adiponectin- and leptin-linked signaling, indicates enhanced cross-talk between adipose tissue and the liver. An enrichment of adipocytokine signaling pathway genes has been reported in liver transcriptomes of early postpartum cows in relation to NEB^46^. The enrichment of the glucagon signaling pathway and insulin resistance pathway in our study is consistent with a metabolic transition toward hepatic glucose production and altered insulin action during early lactation^98,100,104^. Glucagon stimulates hepatic glucose output by activating glycogenesis and gluconeogenesis, which supports the increased glucose demand associated with milk production. Concurrently, reduced systemic insulin sensitivity during early lactation is widely described as an adaptive, homeorhetic feature that helps prioritize glucose utilization by the mammary gland rather than the liver or adipose tissue^105^. In this context, the upregulation of the insulin resistance pathway in our analysis is consistent with this established physiological adaptation. Importantly, the AMPK signaling pathway was also upregulated, highlighting a key energy-sensing mechanism that regulates cellular metabolism. AMPK is activated under conditions of increased AMP:ATP ratios and promotes catabolic pathways such as fatty acid oxidation, while inhibiting anabolic processes like lipogenesis^106^. Its activation in the early postpartum liver is consistent with the energy-deficient state commonly observed in high-producing dairy cows, to restore energy homeostasis. Most of these pathways, including adipocytokine signaling pathway^41,42^, glucagon signaling pathway^41,42,56^, and AMPK signaling pathway^41,42,56,61^, have been reported to be activated in the liver of early postpartum cows.

The transition from late gestation to early lactation imposes substantial metabolic and physiological stress on the liver due to increased demands for gluconeogenesis, lipid mobilization, and overall metabolic reprogramming required to support milk production. The liver must rapidly adapt to meet these demands, and hepatocellular proliferation may be a critical component of this adaptive response. Enhanced hepatocyte proliferation ensures the maintenance of liver functional mass, particularly as cows are susceptible to hepatic lipid accumulation during early lactation under NEB^6,98^. The upregulation of genes governing cell division and chromosome segregation in our study likely reflects a regenerative or compensatory response to increased metabolic workload. Similar patterns of cell cycle gene activation in the liver have been observed in early postpartum Holstein cows, where proliferative signaling was found to be upregulated alongside pathways related to metabolic adaptation and stress response^15^. Additionally, mitosis-related gene activation may be influenced by hormonal and endocrine changes occurring around parturition, and the liver is integrated with growth hormone/IGF-axis physiology that shapes postpartum metabolism^107^.

During the immediate postpartum phase, dairy cows experience a significant increase in energy demand to support the onset of lactation, often while facing insufficient dry matter intake^108^. This imbalance triggers NEB, leading to enhanced liver gluconeogenesis as a compensatory mechanism to maintain blood glucose, critical for milk synthesis^109^. The liver becomes the primary site for glucose production via gluconeogenic pathways, utilizing substrates such as propionate, lactate, and amino acids^110^. The observed upregulation of the TCA cycle pathway in our study supports this physiological shift, as propionate, absorbed from the rumen, is the major glucogenic precursor in ruminants and enters gluconeogenesis via conversion to succinyl-CoA in the TCA cycle^111^. Additionally, enrichment of the pyruvate metabolism is consistent with increased liver flux through pyruvate as an intermediate connecting glycolysis-related inputs to gluconeogenesis and oxidative metabolism. Moreover, glycolytic intermediates may also contribute to biosynthetic processes, including lipid and amino acid metabolism, suggesting a coordinated liver response that supports both energy production and broader metabolic demands during early lactation. Concordantly, GO biological process enrichment supported a shift in intermediary carbon metabolism, with over-representation of broad small-molecule and organic-acid process terms, including small molecule metabolic process and small molecule biosynthetic process, as well as organic acid, carboxylic acid, oxoacid, and monocarboxylic acid metabolic processes, which collectively capture central metabolite transformations that feed into pyruvate and TCA-linked energy metabolism. These findings align with earlier transcriptomic and proteomic studies that have documented the activation of energy-generating and gluconeogenic pathways in the liver during early lactation^23,42,56,112^. The transcriptional upregulation of these metabolic pathways likely reflects a crucial adaptation to the catabolic state of early postpartum cows, underscoring the importance of liver plasticity in sustaining milk production during periods of metabolic challenge. Co-expression gene network analysis further confirmed the activation of the biological processes and KEGG pathways discussed above.

In early postpartum dairy cows, the liver undergoes a marked transition from a more anabolic state to a metabolism geared toward supporting lactation, including substantial hepatic adaptations in glucose and lipid handling^98^. Consistent with this transition, the enrichment of downregulated terms related to carbohydrate and polysaccharide metabolism—including regulation of carbohydrate metabolic process, regulation of glucan biosynthetic process, regulation of glycogen metabolic process and biosynthetic process, and regulation of polysaccharide metabolic process and biosynthetic process—suggests reduced transcriptional emphasis on pathways governing hepatic glycogen and related storage polymers. Instead, the liver prioritizes gluconeogenesis, particularly from non-carbohydrate sources, such as amino acids and glycerol, to sustain plasma glucose levels for the mammary gland^98,113^. This metabolic switch is consistent with reports showing diminished glycogen reserves in the liver of early lactation cows^114^, reflecting both reduced synthesis and active utilization of liver glycogen. The downregulation of stress-response–related terms, including response to ischemia and response to abiotic stimulus, suggests altered transcriptional regulation of hepatic stress-response programs during early lactation. Oxidative stress is widely recognized as a feature of the transition period in dairy cows^115^, and significant changes in hepatic metabolic and signaling gene networks were reported in nutrition-induced ketosis^46^. Beyond carbohydrate-related processes, enrichment of downregulated categories tied to small-molecule metabolism and generation of precursor metabolites and energy (regulation of small-molecule metabolic process; regulation of generation of precursor metabolites and energy) indicates that postpartum hepatic adaptation also involves coordinated changes in multiple intermediary metabolic nodes, rather than a single pathway shift. In parallel, downregulation of lipid biosynthetic process and ketone metabolic process indicates that lipid-related anabolic regulation and ketone-associated regulatory programs are among the transcriptionally responsive modules during this period. This aligns with the established physiology of early lactation in which extensive adipose lipolysis increases hepatic NEFA influx and elevates risk for hepatic lipid accumulation and ketosis-related metabolic remodeling^98,114^, and with transcriptomic evidence that ketosis and early postpartum state reshape hepatic metabolic and signaling networks^46^. The enrichment results also point to broad changes in cell communication and signaling regulation (regulation of signaling; regulation/positive regulation of signaling and signal transduction; regulation/positive regulation of cell communication) and cell population proliferation, indicating that postpartum hepatic adaptation extends beyond metabolism to include regulatory signaling programs and tissue remodeling. The downregulation of the p53 signaling pathway, which is central to apoptosis, DNA repair, and cell cycle arrest, may represent an adaptive response that limits hepatocellular turnover and preserves liver function during metabolic challenges. Consistent with this directionality, inhibition of apoptosis and the p53 signaling pathway was reported in postpartum versus prepartum liver comparison^42^. Finally, the presence of response to ischemia and response to abiotic stimulus among downregulated terms suggests altered stress-response programming during early lactation, a period widely recognized to be associated with systemic oxidative stress and metabolic strain in transition cows^115^ and measurable alterations in hepatic mitochondrial function in early lactation^116^. Notably, enrichment of the ovulation cycle term among downregulated hepatic DEGs may reflect the broader postpartum physiology in which negative energy balance and metabolic load are associated with delayed resumption of ovarian cyclicity and reduced reproductive efficiency^117,118^.

This study has several important limitations. First, the small sample size (n = 6) limits statistical power, increases sensitivity to inter-individual variability, and constrains population-level inference; accordingly, these results should be interpreted as exploratory and hypothesis-generating rather than definitive. Second, the two-time-point design (D−21 and D+7) provides only a transcriptomic snapshot and may not capture transitional dynamics occurring between or outside these windows. Third, gene-expression changes were not directly integrated with phenotypic or metabolic measures (e.g., plasma NEFA/BHBA, liver triglyceride content, milk production traits), which limits the ability to link transcriptional shifts to functional outcomes. Finally, due to limited remaining RNA, targeted qPCR validation was not performed; future work in larger, independent cohorts with denser temporal sampling and integrated metabolic/performance phenotyping will be needed to confirm key differentially expressed genes and refine biological interpretation.

In summary, this study provides a well-documented liver transcriptomic dataset from U.S. Holstein cows sampled at D−21 prepartum and D+7 postpartum under a clearly defined management and dietary context. We observed coordinated postpartum hepatic reprogramming, with increased expression of genes and pathways related to lipid handling, energy metabolism and gluconeogenesis, and decreased expression of glycogen synthesis and select immune/transport functions, consistent with transition-period adaptation to lactation-associated energy demands under negative energy balance. Given the limited sample size, two-time-point design, absence of paired metabolic/performance phenotypes, and lack of qPCR validation, the findings should be interpreted as exploratory and hypothesis-generating. Future studies with larger cohorts, finer temporal resolution, independent validation, and integration of transcriptomic, metabolic, and production traits will be required to establish robust links between hepatic gene regulation and transition-period health and performance.

## Supplementary Information

Supplementary Information 1.

Supplementary Information 2.

Supplementary Information 3.

Supplementary Information 4.

Supplementary Information 5.

Supplementary Information 6.

Supplementary Information 7.

Supplementary Information 8.

Supplementary Information 9.

Supplementary Information 10.

## Data Availability

The data that support the results of this research are available within the article and its Supplementary Information files. The datasets generated and analyzed during the current study are available in the SRA repository under accession PRJNA1321943 at https://dataview.ncbi.nlm.nih.gov/object/PRJNA1321943.
