# Supplementary figures and images for: Liver transcriptome dynamics in Holstein cows during the periparturient transition
Source: Sci Rep. 2026 Apr 4;16:16227. doi: 10.1038/s41598-026-46925-9 (PMC13201770; doi:10.1038/s41598-026-46925-9)

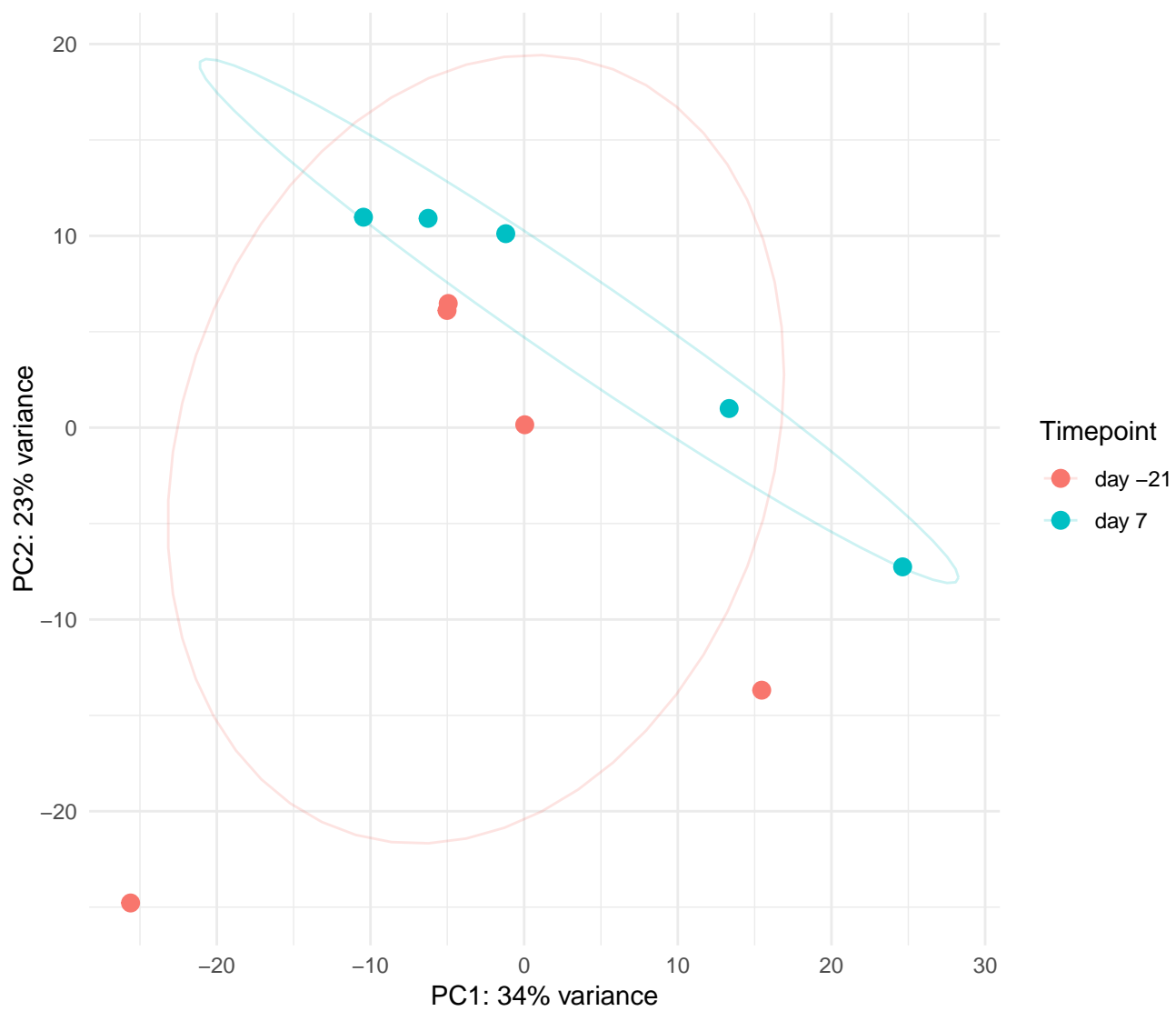

Supplement: Supplementary file 1 — Supplementary Information 1. [file 41598_2026_46925_MOESM1_ESM.pdf]

## Raw Reads

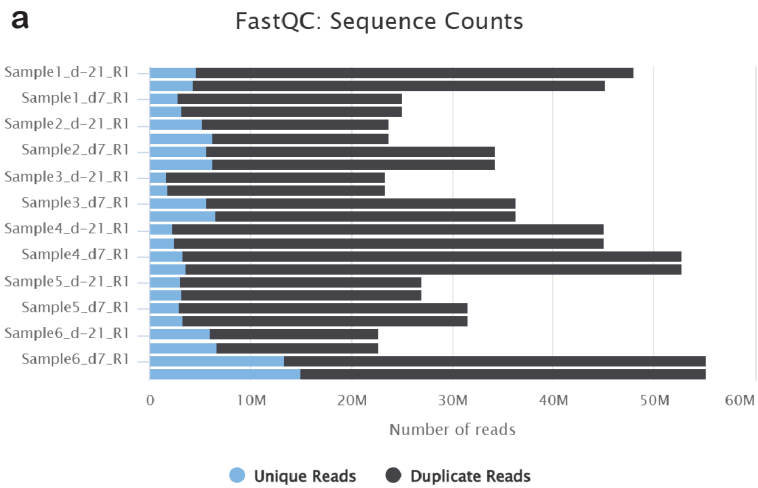

## Clean Reads

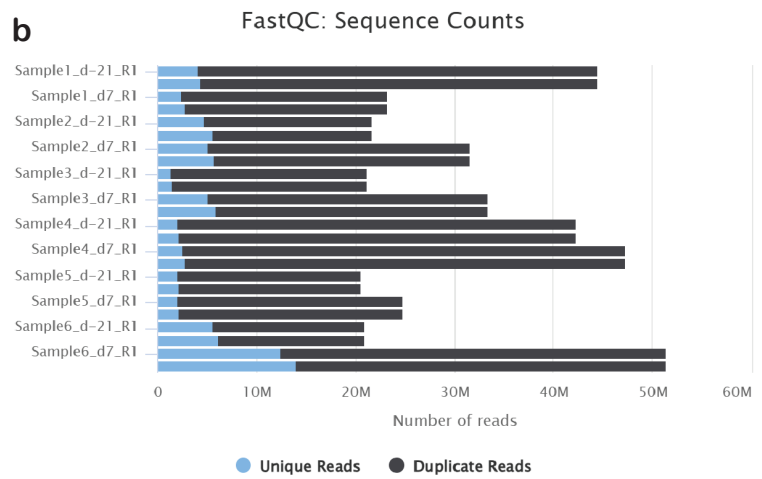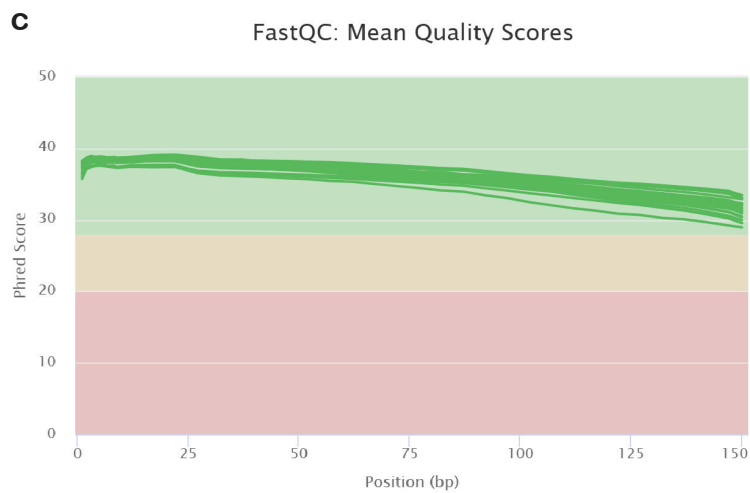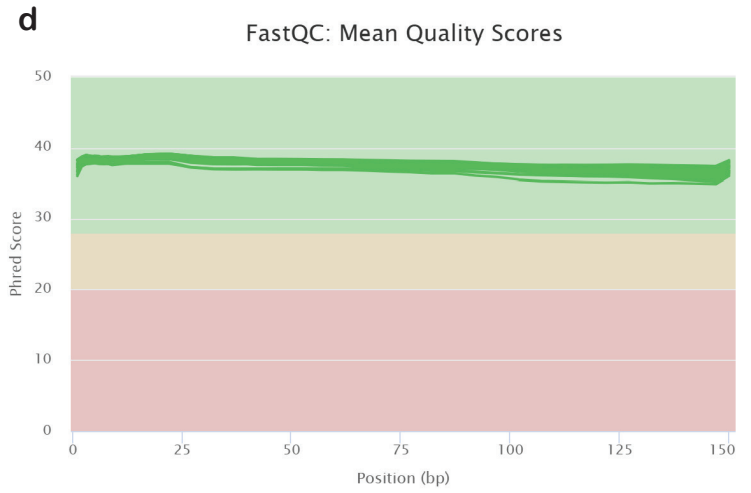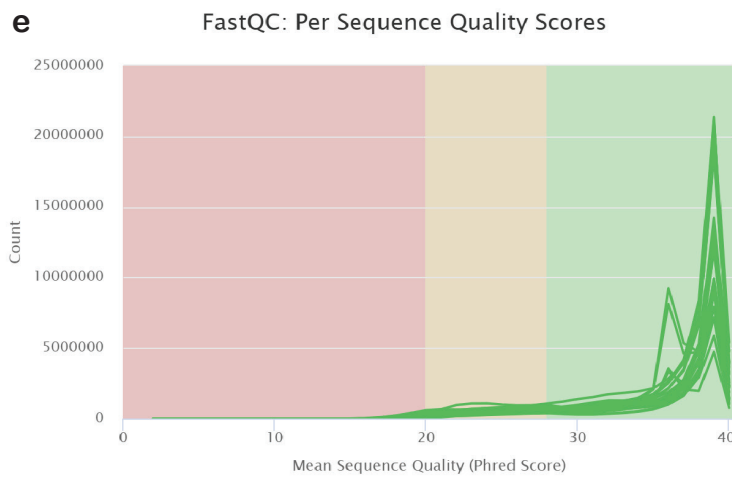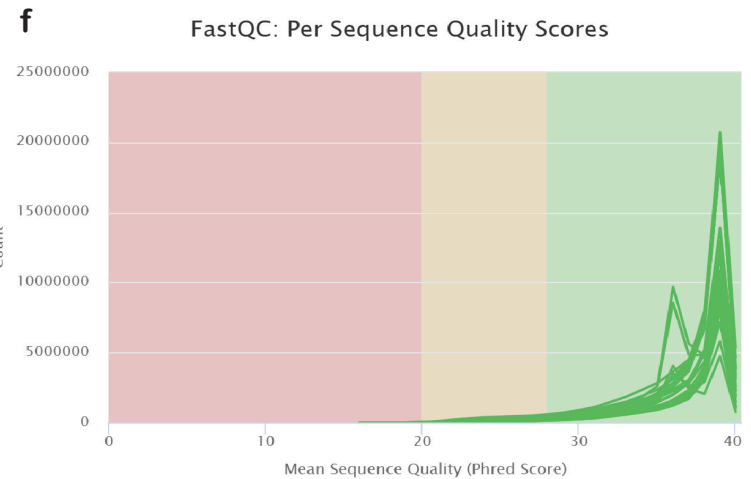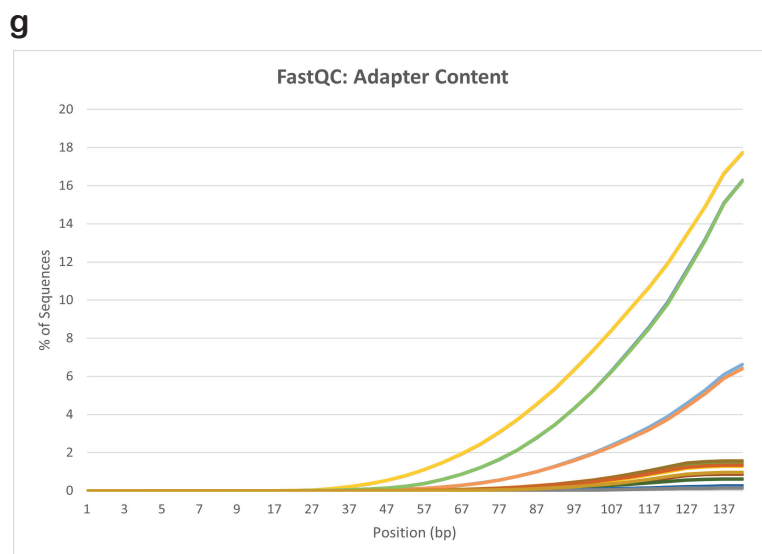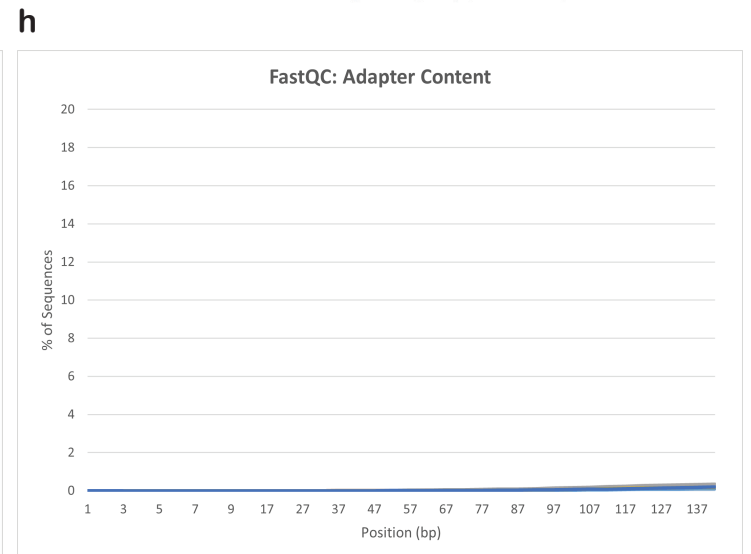

Supplement: Supplementary file 2 — Supplementary Information 2. [file 41598_2026_46925_MOESM2_ESM.pdf]
