# Supplementary material for: Liver transcriptome dynamics in Holstein cows during the periparturient transition
Source: Sci Rep. 2026 Apr 4;16:16227. doi: 10.1038/s41598-026-46925-9 (PMC13201770; doi:10.1038/s41598-026-46925-9)
Supplement: Supplementary file 10 — Supplementary Information 10. [file 41598_2026_46925_MOESM10_ESM.docx]

**Supplementary Tables**

**Table S1** Quality assessment information of raw and clean RNA-seq reads.

**Table S2** Mapping information of RNA-seq data.

**Table S3** Differentially expressed genes (DEGs) in liver at 7 days post-calving in relative to 21 days pre-calving, ranked by ascending adjusted *P*-value.

**Table S4** List of enriched GO biological process (a), molecular function (b), and cellular component (c) terms in the up-regulated genes.

**Table S5** List of enriched GO biological process (a), molecular function (b), and cellular component (c) terms in the down-regulated genes.

**Table S6** List of enriched GO biological process (a), molecular function (b), and cellular component (c) terms in the genes of the significant red module.

**Table S7** Comparison of major periparturient liver transcriptome RNA-seq studies.

**Supplementary Figures**

**Fig. S1** Principal component analysis (PCA) plot of 11 samples used for downstream analysis. This plot was created using normalized counts generated by DESeq2.

**Fig. S2** Quality assessment reports of raw and clean RNA sequenced reads. **a** Unique and duplicate read counts for raw reads. **b** Unique and duplicate read counts for clean reads. **c** Mean quality scores for raw reads. **d** Mean quality scores for clean reads. **e** Per sequence quality scores for raw reads. **f** Per sequence quality scores for clean reads. **g** Adapter content in raw reads. **h** Adapter content in clean reads.
